# Serine racemase deletion alters adolescent social behavior and whole-brain cFos activation

**DOI:** 10.3389/fpsyt.2024.1365231

**Published:** 2024-06-24

**Authors:** Stephanie E. Brown, Ziyi (Zephyr) Wang, Emily L. Newman, Elif Engin, Sabina Berretta, Darrick T. Balu, Oluwarotimi O. Folorunso

**Affiliations:** ^1^ Division of Basic Neuroscience, Translational Psychiatry Laboratory, McLean Hospital, Belmont, MA, United States; ^2^ Division of Basic Neuroscience, Stress Neurobiology Laboratory, McLean Hospital, Belmont, MA, United States; ^3^ Department of Psychiatry, Harvard Medical School, Boston, MA, United States; ^4^ Division of Depression and Anxiety Disorders, Neurobiology of Fear Laboratory, McLean Hospital, Belmont, MA, United States; ^5^ Division of Basic Neuroscience, Translational Neuroscience Laboratory, McLean Hospital, Belmont, MA, United States

**Keywords:** D-serine, social novelty, cFos, adolescence, brain networks

## Abstract

**Background:**

Neurodevelopmental disorders (NDDs) can cause debilitating impairments in social cognition and aberrant functional connectivity in large-scale brain networks, leading to social isolation and diminished everyday functioning. To facilitate the treatment of social impairments, animal models of NDDs that link N- methyl-D-aspartate receptor (NMDAR) hypofunction to social deficits in adulthood have been used. However, understanding the etiology of social impairments in NDDs requires investigating social changes during sensitive windows during development.

**Methods:**

We examine social behavior during adolescence using a translational mouse model of NMDAR hypofunction (SR-/-) caused by knocking out serine racemase (SR), the enzyme needed to make D-serine, a key NMDAR coagonist. Species-typical social interactions are maintained through brain-wide neural activation patterns; therefore, we employed whole-brain cFos activity mapping to examine network-level connectivity changes caused by SR deletion.

**Results:**

In adolescent SR-/- mice, we observed disinhibited social behavior toward a novel conspecific and rapid social habituation toward familiar social partners. SR-/- mice also spent more time in the open arm of the elevated plus maze which classically points to an anxiolytic behavioral phenotype. These behavioral findings point to a generalized reduction in anxiety-like behavior in both social and non-social contexts in SR-/- mice; importantly, these findings were not associated with diminished working memory. Inter-regional patterns of cFos activation revealed greater connectivity and network density in SR-/- mice compared to controls.

**Discussion:**

These results suggest that NMDAR hypofunction – a potential biomarker for NDDs – can lead to generalized behavioral disinhibition in adolescence, potentially arising from disrupted communication between and within salience and default mode networks.

## Introduction

Neurodevelopmental disorders (NDDs) such as schizophrenia (SZ) or autism spectrum disorder (ASD) share a range of social cognitive deficits that become apparent during development and can predict symptom severity in adulthood. To understand and treat social deficits in adulthood, it is important to examine the neurobiological changes that support social development during childhood and adolescence. In NDDs, the lack of social motivation or altered internal states may limit or enhance social play and dysregulate sociability during adolescence (1). For example, children with ASD may have problems initiating social interaction (2), children likely to develop schizophrenia may have issues inferring social cues (3), while children with William-Beuren syndrome (WBS) may show an increase in social drive (4). The ability to engage in productive social interactions depends on the solidification of essential neural pathways during adolescence, a sensitive period of development coinciding with peaks in synaptic pruning (5). Understanding the molecular and neural basis of social behavior and its disruption in NDDs will be crucial for developing interventions that can improve outcomes for these disorders. Patients with SZ or ASD experience symptoms tied to deficits in functional connectivity within the large-scale brain networks that uphold sociability including the default mode network (DMN; self-reflection) and salience network (SN; external or internal stimuli (1). Brain regions important for social functioning are high represented in the DMN (6). In adolescence, changes in DMN are concomitant with the severity of social impairment (6).

Adaptive and effective neuronal communication relies heavily on excitation-inhibition (E-I) balance (7), and significant evidence points to developmental E-I imbalances as an underlying cause of NDDs. Maintenance of E-I balance relies upon amino acid neurotransmission including the careful regulation of excitatory glutamatergic signaling via *N*-methyl-D-aspartate receptors (NMDARs). Genome-wide association studies (GWAS) in humans with schizophrenia or ASD have implicated genes and pathways important for glutamatergic signaling (8, 9) in these NDDs. Preclinical rodent studies that pharmacologically ablate NMDAR-mediated neurotransmission yield notable social deficits and reduced inhibitory neurotransmission in the medial PFC (10). Deletion of NMDARs in corticolimbic (striatum, cortex, hippocampus) interneurons during adolescence – but not adulthood – promotes aberrant sociability (11–13). In sum, these findings suggest a developmental role of NMDAR activity and point to E/I imbalance during development as a potential mechanism by which NDDs yield persistent dysregulation within the functional brain networks that uphold adaptive sociability.

Binding of glutamate and a coagonist is required for NMDAR-mediated signaling and for maintenance of E/I balance. D-serine has gained recognition as the primary NMDAR coagonist in cortico-limbic pathways that comprise the DMN and SN (14). L-serine is synthesized by astrocytes and then transported by neurons to be converted to D-serine by serine racemase (SR). As such, mice harboring a constitutive SR knockout (SR-/-) exhibit significant deficits in NMDAR-mediated signaling and plasticity in the hippocampus and dentate gyrus (15–21).

In our previous work, we demonstrated that adult SR-/- mice exhibit social deficit and impairments in social task-elicited frontal cortical gamma power, which is important for E/I balance (22). We also showed that juvenile SR-/- mice display fewer cortical interneurons and reduced prelimbic inhibitory neurotransmission (23). We observed a marked a spatio-temporal developmental increase in SR expression in the prefrontal cortex, amygdala, and nucleus accumbens areas comprising the brain networks that reinforce adaptive forms of sociability (24). Administration of D-serine rescued social deficits in NDD models with NMDAR hypofunction (25). While these findings demonstrate a critical role for D-serine signaling during social interactions in adulthood, it remains unclear whether the effects of constitutive SR-/- can be traced back to developmental social deficits during adolescence. As such, in the present work, we examine how adolescent social interactions are affected by SR-knockout-induced glutamatergic hypofunction, a model of NDD-associated E/I imbalance.

## Methods

### Animal behavior

#### Animals

Homozygous WT or SR-/- offspring of SR+/- parents served as experimental animals. Heterozygous offspring were also employed to examine potential gene-dosing effects in a subset of pilot studies (see Supplement). Mice were weaned at post-natal day (PND) 21 and then grouped-housed with same-sex littermates (n=2–5/cage). Mice were maintained on a 12h light/dark cycle in a temperature (22C)- and humidity-controlled vivarium. Animals were given access to food and water ad libitum. All experiments complied with ethical regulations for animal testing and research. The McLean Hospital Institutional Animal Care and Use Committee approved all animal procedures. All behavioral tests were conducted with ambient lighting at approximately 10 lux and were recorded using Ethovision XT 15 (Noldus).

#### 3-Chamber sociability and social novelty testing

Sociability and social novelty preference tests were examined in a 3-chamber apparatus using protocols adapted from DeVito et al. (26). SR-/- and WT littermates (PND 27–30; *n* = 14 males/genotype; *n* = 5–7 females/genotype) were habituated to the test room for at least 60 minutes. After habituating to the testing room, each mouse habituated to the entire apparatus for five minutes and then to just the center chamber for five minutes.

Following 10-minute apparatus habituation, mice were tested for sociability, or pereference for a social vs. non-social stimulus. An unfamiliar age-matched WT stimulus mouse was placed in a small wire mesh cage on one side of the chamber while an unfamiliar object was placed in the small wire mesh cage within the opposite chamber. Social and non-socail stimulus sides were counterbalanced such that half of experimental mice received the social stimulus in the far-right chamber and half received the social stimulus in the far-left chamber of the 3-chamber apparatus. The sociability test commenced when the experimental animal was released from the center chamber and permitted to explore the entire apparatus for 10 minutes. After 10 minutes, the experimental mouse was ushered back to the center chamber. Five-minute evaluations of social novelty preference were conducted thereafter to examine how much time each experimental mouse spent investigating the now-familiar social stimulus mouse that was previously presented during the sociability test (mouse 1) vs. time spent engaging with an unfamiliar social stimulus (mouse 2). Social and non-social investigation were quantified as time spent within a circular zone extending 3-cm past the each small cage (Noldus Ethovision XT 15). The sociability preference index was calculated by subtracting the time spent with the object from the time spent with the stimulus mouse (novel mouse “1”) and then dividing that value by the combined time spent interacting with both the mouse and the object. The social novelty preference index was calculated by subtracting the time spent with the familiar mouse (novel mouse “1”) from the time spent with the novel mouse (novel mouse “2”) and then dividing that value by the combined time spent interacting with both mice. (27).

#### Social memory

Social memory was tested in male mice (PND30; *n* = 7/genotype; 28). In brief, mice were habituated to the testing room in their home cages for at least 60 minutes. Experimental mice were then placed in the center of the empty testing arena to habituate for five minutes. An unfamiliar age-matched stimulus mouse in a small wire mesh cage was placed in the center of the arena and the experimental animal was permitted to investigate for five minutes. Thirty minutes later, the experimental mouse was permitted to investigate the same caged social stimulus animal for an additional five minutes. The time the experimental mouse spent within the 3-cm interaction zone was quantified (Noldus Ethovision XT 15).

#### Y-maze

Spontaneous alternations in a Y-maze task were measured to assess spatial and working memory. The Y-maze used in the present study consisted of three arms (A, B, and C), all (83 x 66.5 cm), and an equilateral triangular center. Male mice (PND 33–38; *n* = 7–9/genotype) habituated to the testing room for at least 30 minutes. Subsequently, mice were placed in arm “A,” facing the center of the maze and allowed to move freely through the maze for five minutes. A spontaneous alternation in this task consists of consecutive entries into each of the three arms in the maze, representing the most efficient exploratory strategy. The alternation percentage was calculated by dividing the number of alternations by the number of total possible alternations. Total possible alternations were calculated by subtracting two from the total number of arm visits.

#### Elevated plus maze (EPM)

We assessed anxiolytic behavior using the standard EPM appartus, which consisted of two ‘closed’ and two ‘open’ arms (12 L x 2.5 W inches) crossed at a ninety-degree angle and elevated (32.5 inches) above the ground. Mice were placed in the center facing the open arm to start the five-minute trial. Before testing, mice (males PND 35; *n* = 10–13 per genotype) were habituated to the testing room for at least 30 minutes. The ratio of time spent in the open arms was calculated by dividing the total time spent in the open arms by the total time spent in the closed arms.

### cFos brain-wide imaging and analysis

#### Tissue preservation and clearing, immunolabeling, and imaging

Paraformaldehyde-fixed brains were preserved using SHIELD reagents (LifeCanvas Technologies) following the manufacturer’s instructions (29). Samples underwent LifeCanvas Technologies Clear+ de-lipidation and immunolabeling (cFos abcam ab214672; NeuN Encor MCA) using eFLASH (30) technology, which integrates stochastic electrotransport (31) and SWITCH (32) using a SmartBatch+ device (LifeCanvas Technologies). After immunolabeling, samples were incubated in 50% EasyIndex (RI = 1.52, LifeCanvas Technologies) overnight at 37 degrees C, followed by 1-day incubation in 100% EasyIndex for refractive index matching. After index matching, the samples were imaged using a SmartSPIM light sheet microscope using a 3.6x (0.2 NA) using 488 and 642 nm lasers (LifeCanvas Technologies).

#### Atlas registration

Samples were registered to the Allen Brain Atlas (Allen Institute: https://portal.brain-map.org/) using an automated process (alignment performed by LifeCanvas Technologies). A NeuN channel for each brain was registered to an average NeuN atlas (generated by LCT using previously registered samples). Registration was performed using successive rigid, affine, and b-spline warping algorithms (SimpleElastix: https://simpleelastix.github.io/).

#### Cell detection

Automated cell detection was performed by LifeCanvas Technologies using a custom convolutional neural network created with the Tensorflow python package (Google). The cell detection was performed by two networks in sequence. First, a fully-convolutional detection network (https://arxiv.org/abs/1605.06211v1) based on a U-Net architecture (https://arxiv.org/abs/1505.04597v1) was used to find possible positive locations. Second, a convolutional network using a ResNet architecture (https://arxiv.org/abs/1512.03385v1) was used to classify each location as positive or negative. Using the previously calculated Atlas Registration, each cell location was projected onto the Allen Brain Atlas in order to count the number of cells for each atlas-defined region.

#### Brain functional connectivity network

We analyzed 196 regions spanning iso-cortex, cortical plate, cortical subplate, striatum, pallidum, thalamus, hypothalamus, midbrain, pons, and medulla.

We assessed the network density, which indicates the level of interconnectedness among the brain regions by measuring the proportion of actual connections relative to the total potential connections (33). Apart from looking at all the regions in an unsupervised approach, we conducted independent analyses focused on brain regions comprising functional brain networks including the social, salience, lateral cortical, and default mode networks (34–36). We constructed a binary adjacency matrix for network analysis, values in the resulting matrix were set to 1 if they exceeded the chosen threshold, and 0 otherwise.

#### Statistics

We used GraphPad Prism (San Diego, California, USA) and Excel for the statistical analyses performed on the behavioral assays. For comparisons between groups, we used either unpaired two-tailed t-tests, two-way analyses of variance (ANOVA), or repeated measure two-way ANOVA. One-sample t-tests were used to compare preference indexes to a null hypothesis. The bar graphs depict group mean  ± SEM.

For cFos density data, we used Python SciPy (37) and statsmodels (38) packages for statistical analysis. We conducted Shapiro-Wilk test to ensure the normal distribution within brain regions (**Supplementary Table 1**) as well as at the whole brain level, and performed multiple unpaired t-tests with Benjamini and Hochberg FDR correction. To understand the potential co-activation patterns and address the uneven sample size between groups, we calculated non-parametric Spearman’s rank correlation coefficients (ρ values) between the cFos density measurements using seaborn package in Python (39). To identify connections that exhibit a strong level of coactivation, the adjacency matrix was used a threshold based on the absolute effect size (|ρ| ≥ 0.8) (40). We also applied p-value with correction using Benjamini and Hochberg false discovery rate (5% FDR) as a threshold to investigate the significantly correlated regions.

## Results

### Adolescent SR-/- mice preferentially interact with novel social partners

**Figure 1:**
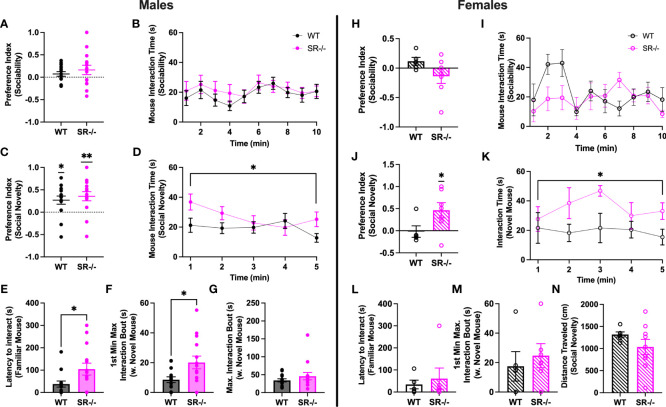
Male and female adolescent (PND27–30) SR-/- mice show an increase in social interaction with a novel mouse. **(A, H)** Graphs show the sociability preference indexes. **(B, I)** Graphs show the time in seconds spent interacting with the stimulus mouse in the sociability trial. **(C, J)** Graphs show the social novelty preference indexes and the results of one-sample t-tests comparing the observed means to a hypothetical mean. **(D, K)** Graphs show the time in seconds spent interacting with the novel mouse in the social novelty trial. **(E, L)** Graphs show latency to interaction with the familiar mouse in the social novelty trial. **(F, M)** Graphs show the maximum interaction bout with the novel mouse in the first minute of the social novelty trial. **(G, N)** Graphs show the distance traveled by each genotype in the social novelty trial. Males: *n* = 14 (both genotypes); females: WT: *n* = 5; SR-/-: *n* = 7. *p < 0.05, **p < 0.01. One-sample t-test, unpaired t-test, and repeated measure ANOVA.

Neither male nor female adolescent SR-/- and WT mice preferred social stimuli over non-social stimuli (**Figures 1A, B, H, I**). WT and SR-/- males and SR-/- females preferred to interact with a novel social stimulus as compared to a familiar social stimulus (**Figure 1C**; WT males: t[13] = 2.943, p = 0.0114; SR-/- males: t[13] = 3.461, p = 0.0042), in the females, SR-/- mice showed this preference (**Figure 1J**; t[6] = 2.686, p = 0.0362). Examining time-binned behavior over the course of the social novelty test revealed that male and female adolescent SR-/- mice spent more time with a novel social partner compared with their WT counterparts (**Figure 1D**; males: F[1,27] = 5.097, Hedge’s g = 0.126, η^2^_p_ = 0.061, p = 0.032; **Figure 1K**; females: F[1, 9] = 5.636, Hedge’s g = 0.248, η^2^_p_ = 0.254, p = 0.042). Although we did not observe a social preference in the WT female mice, the interaction time with the novel mouse was similar to those observed in the male WT mice. Exploring social interaction bouts revealed that male SR-/- mice took significantly longer to interact with the familiar mouse (**Figure 1E**; t[27] = 2.622, p = 0.032) and had longer bouts of continuous interaction with the novel mouse (**Figure 1F**; t[27] = 2.520, p = 0.018) within the first minute of the social novelty test. These differences were not seen in the female mice (**Figures 1L, M**). There were no differences in distance traveled between the genotypes (**Figures 1G, N**) or any apparent gene-dosing effects when heterozygous mice were included in pilot analyses of WT, SR+/-, and SR-/- mice (**Supplementary Figure 1**). As such, we chose to examine only WT and SR-/- mice in subsequent experiments. In females, only the SR-/- displayed a significant social novelty preference (**Supplementary Figure 1G**; t(6) = 2.87, p = 0.03). However, similar to the males, there were no significant differences between the genotypes in the time spent with the novel mouse (**Supplementary Figure 1H**).

### Adolescent male SR-/- mice exhibit deficient sociability during repeated interaction with the same partner

**Figure 2:**
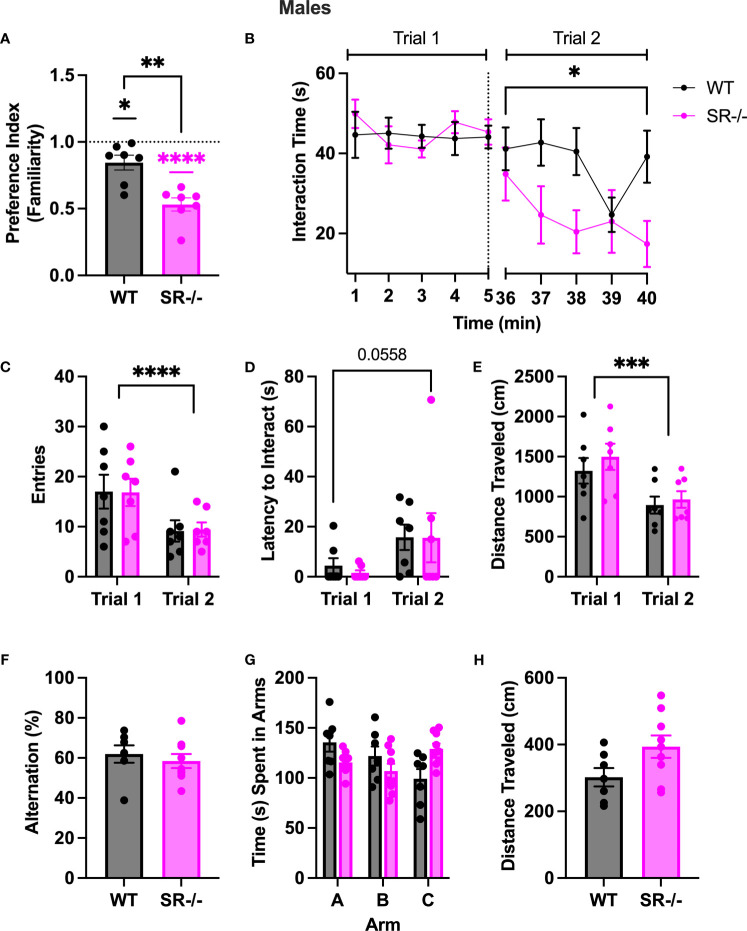
Adolescent (PND30) male SR-/- mice spend less time with a familiar mouse in a two-trial sociability test. **(A)** Graph shows familiarity preferences in both genotypes **(B)** Graph shows the time in seconds spent interacting with a stimulus mouse (trial 1) and the same mouse after a 30-minute interval (trial 2). **(C)** Graph shows the number of entries to the interaction zone with the stimulus mouse in both trials. **(D)** Graph shows the latency, in seconds, the first entry of the interaction with the stimulus mouse in both trials. **(E)** Graph shows the distance traveled, in centimeters, of the test mouse in trial 1 and trial 2. **(F)** Graph shows the percentage of successful alternations completed by each genotype in the Y-maze task. **(G)** Graph shows the time, in seconds, spent in each arm of the Y maze. **(H)** Graph shows the distance traveled in the Y-maze. Two trial sociability test: WT n = 7 for both genotypes; Y-Maze: WT: *n* = 7; SR/- *n*: 9. *p < 0.05, **p < 0.01, ****p < 0.0001. One-sample t-test, unpaired t-test, and repeated measure ANOVA.

To determine whether the preference for a novel social stimulus in SR-/- adolescent mice was due to impaired social recognition or social memory, we performed a two-trial social memory test. Notably, in trial two, both groups showed: (1) a lack of familiarity preference, calculated by using a one-sample t-test to compare the mean preference against a hypothetical mean of one (**Figure 2A**; WT: t[6] =15.15, p < 0.001; SR-/-: t[6] = 10.72, p < 0.001); (2) expedited social habituation as supported by diminished social interaction time with the familiar social partner over time compared to WT controls (**Figure 2B**; F[4.737, 56.84] = 6.194, Hedge’s g = 0.334, η^2^_p_ = 0.340, p < 0.0002); and fewer zone entries (**Figure 2C**; F[1, 12] = 20.77, Hedge’s g = 0.592, η^2^_p_ = 0.634, p = 0.0007); (3) a trend toward an increased latency to interact with the stimulus mouse (**Figure 2D**; F[1, 12] = 4.482, Hedge’s g = 0.253, η^2^_p_ = 0.272, p = 0.0558) and; (4) a reduction in overall distance traveled (**Figure 2E**; F[1, 12] = 28.44, Hedge’s g = 0.655, η^2^_p_ = 0.703, p = 0.0002). In accordance, the SR-/- mice had a significantly lower familiarity preference than the WT mice (**Figure 2A**; t[12] = 4.204, p = 0.0012) and spent significantly less time with the stimulus mouse in the second trial than the WT mice (**Figure 2B**; F [9, 108] = 2.291, Hedge’s g = 0.159, η^2^_p_ = 0.160, p = 0.025).

### Intact spatial working memory in adolescent SR-/- and WT mice

To determine if the differences in social novelty preference and repeated social interaction were due to differences in spatial working memory between WT and SR-/- adolescent mice, we then subjected both genotypes to a spontaneous alternation Y-maze task. No difference in alternation percentage, time spent in the arms, or distance traveled was observed between the SR-/- to WT mice (**Figures 2F–H**).

### Increased exploratory behavior in SR-/- adolescent males

**Figure 3:**
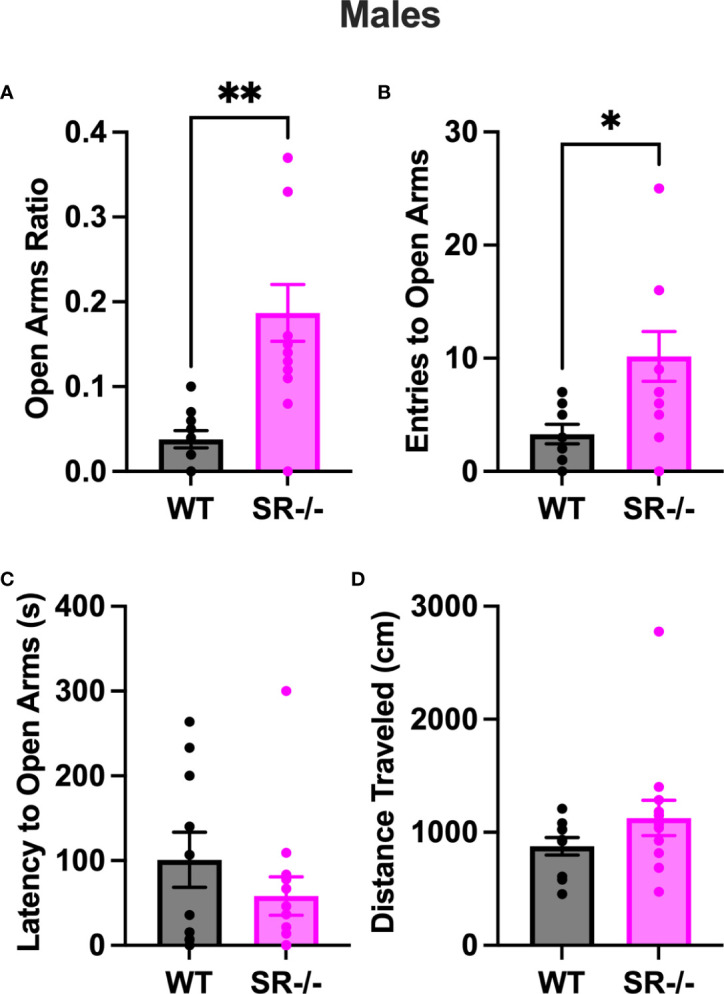
SR-/- adolescent male mice show an increase in exploratory behavior. **(A)** Graph shows ratio of time spent in the open arms of an elevated plus maze. **(B)** Graph shows the number of entries to the open arms of the maze. **(C)** Graph shows the latency in seconds before first entry to the open arms. **(D)** Graph shows the distance traveled in the maze. WT: *n* = 10, SR-/-: *n* = 13. *p < 0.05, **p < 0.01. Unpaired t-test.

Compared to the WT, SR-/- males spent a significantly higher ratio of time in the open arms of the EPM (**Figure 3A**, t[21] = 3.784, p = 0.011). Additionally, they also displayed a significantly higher number of entries to the open arms (**Figure 3B**, t[21] = 2.605, p = 0.0165). We did not observe any differences between genotypes in the latency to the open arms (**Figure 3C**) and the distance traveled in the arena (**Figure 3D**). The increase in time spent in and entries to the open arms was only seen in the SR-/- and not SR +/- mice (**Supplementary Figures 2A, B**; F[2, 32] = 6.698, Hedge’s g = 0.286, η^2^_p_ = 0.295, p = 0.0037, F[2, 32] = 3.824, Hedge’s g = 0.188, η^2^_p_ = 193, p = 0.0324).

### Brain-wide social novelty-induced activity mapping in SR-/- and WT mice

**Figure 4:**
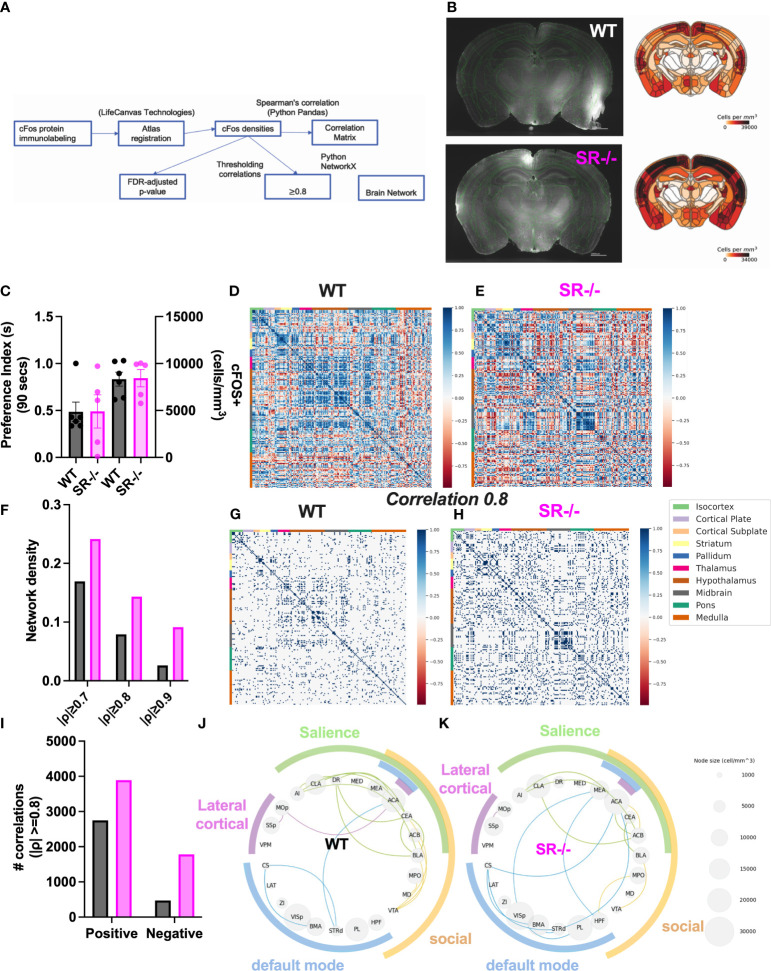
Increase in cFos network density in male SR-/- mice. **(A)** Whole-brain cFos analysis strategy **(B)** Representative cFos staining and heatmaps from WT and SR-/- sections **(C)** Total cFos densities and preference for a novel social stimulus animal in the three-chambered social interaction paradigm in adolescent (PND28) WT (grey) and SR-/- (magenta) mice. **(D, E)** All correlations between 196 brain regions. **(F)** Network densities with increasingly stringent thresholding **(G, H)** Correlations that survive moderately stringent thresholding (||0.8 threshold in WT and SR-/- respectively. **(I)** Number of positive and negative correlations after Spearman’s |ρ| ≥ 0.8 thresholding. **(J, K)** cFos analyses in functional brain networks including the Default Mode (blue), Salience (green), Social (yellow) and Lateral cortical (purple) networks. Circle plots showing the correlations between regions within each functional network (|ρ| ≥ 0.8). Node size indicates regional cFos density (WT: *n* = 6, SR-/-: *n* = 5).

**Figure 5:**
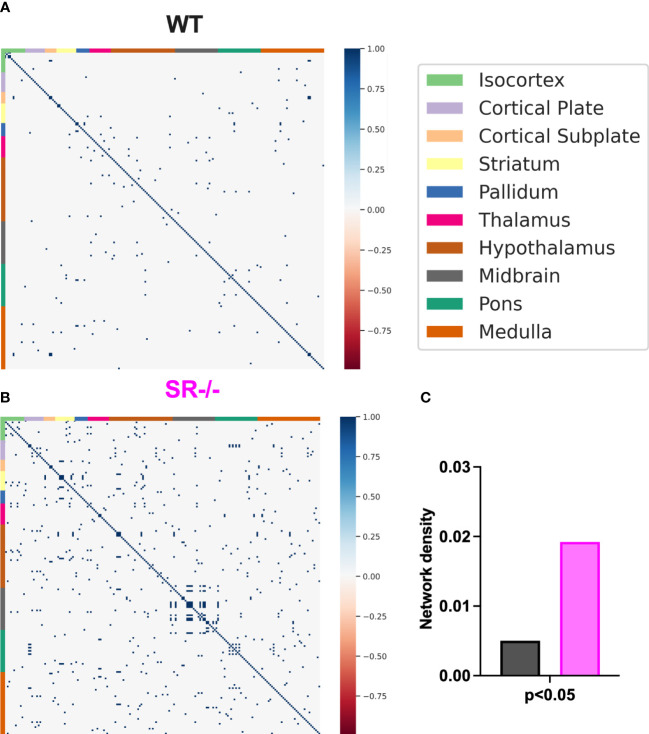
Correlations and network density in male adolescent mice after adjusting for the number of brain regions. **(A, B)** Correlation matrices after adjusting for the 196 brain regions. **(C)** WT (*n*=6) and SR-/- (*n*=5) network density. Spearman’s rank correlation coefficients with FDR correction.

To assess the impact of SR deletion on brain network activation, we utilized whole-brain cFos expression induced during the first 90 seconds of the social novelty task. We hypothesized that we would see cFos density changes in social brain networks. We constructed a comprehensive brain network comprising of 196 brain regions (**Figure 4A**) and generated correlation matrices using different thresholds and the adjusted p-value. We show images and heatmaps for cFos detection in WT and SR-/- sections (**Figure 4B**). **Figure 4C** shows the interaction with the novel mouse used for c-Fos whole brain imaging and the total cFos densities for the 196 brain regions. There was a distinctive pattern of cFos expression in SR-/- compared to WT (**Figures 4D, E**) mice. To determine if these correlations are not by chance, we applied multiple effect size thresholds. We show an increase in the network density (# correlation/# possible correlations) using multiple (0.7, 0.8, 0.9) thresholds (**Figure 4F**). We next plotted the correlation matrices using 0.8 as an exploratory threshold due to the number of correlations identified and sample size (**Figures 4G, H**). We show that both the positive and negative correlated areas are increased after thresholding in the SR-/- compared to the WT brains (**Figure 4I**). We converted the correlation matrix to an adjacency matrix (conversion to a binary number based on |ρ| ≥ 0.8) to develop functional connectivity plots. We analyzed the default mode, salience, social, and lateral cortical networks independently (**Figures 4J, K**). There was an increase in connections and changes in the predicted connectivity in SR-/- in the default network and a decrease in the salience network compared to WT brains (**Figures 4J, K**; **Table 1**). After adjusting for both the number of brain regions (**Figures 5A, B**), we still observed an increase in network density (**Figure 5C**) in SR-/- compared to WT brains. For cFos cell density in each brain regions, we see a significant increase in ventral tegmental area (**Supplementary Figure 3C**, t[8.909] = 3.372, p = 0.008347, q = 0.082640). There were no significant changes in other brain regions within these networks after FDR correction (**Supplementary Figures 3A, B, D**).

**Table 1:** Correlations between brain regions within the salience, social, default mode and lateral cortical networks.

WT				SR-/-			
region 1	region 2	ρ	network	region 1	region 2	ρ	network
CEA	BLA	1	social	CEA	BLA	0.9	social
MD	MPO	0.88571429	social	ACB	ACA	0.9	social
VTA	BLA	0.82857143	social	VTA	BLA	0.9	social
VTA	CEA	0.82857143	social	MEA	HPF	0.8	social
VTA	ACB	0.94285714	social	MPO	HPF	0.8	social
ACA	STRd	0.82857143	default	STRd	ACA	0.9	default
STRd	CS	0.82857143	default	BMA	STRd	0.9	default
CS	BMA	0.94285714	default	VISp	PL	0.9	default
				CS	ACA	0.9	default
				CS	STRd	1	default
				CS	BMA	0.9	default
				MEA	HPF	0.8	default
				MEA	VISp	0.8	default
BLA	AI	0.82857143	salience	ACB	AI	0.9	salience
CLA	AI	0.82857143	salience	CLA	ACB	0.9	salience
BLA	CEA	1	salience	CEA	BLA	0.9	salience
CEA	AI	0.82857143	salience	ACA	AI	1	salience
CEA	BLA	1	salience	ACA	ACB	0.9	salience
CEA	CLA	1	salience				
DR	BLA	0.94285714	salience				
DR	CLA	0.94285714	salience				
DR	CEA	0.94285714	salience				
ACA	DR	0.88571429	salience				
Mop	ACA	0.88571429	lateral cortical	MOp	SSp	1	lateral cortical

The table shows more strong correlations (|ρ| ≥ 0.8) in default network for SR-/- than for WT and more strong correlations in salience network for WT than for SR-/-. Salience network is comprised of: AI, agranular insular are; BLA, basolateral amygdalar nucleus; CEA, central amygdalar nucleus; MEA, medial amygdalar nucleus; MED, medial group of dorsal thalamus; DR, dorsal nucleus raphe; CLA, claustrum. Social network is comprised of HPF, hippocampal formation; ACA, anterior cingulate area; PL, prelimbic area; VTA, ventral tegmental area; MD, mediodorsal nucleus of thalamus; ACB, nucleus accumbens; MPO, medial preoptic area; MEA, CEA, and BLA. Default mode network is comprised of STRd, striatum dorsal region; VISp, primary visual area; BMA, basomedial amygdala; LAT, lateral group of the dorsal thalamus; ZI, zona incerta; CS, superior central nucleus raphe, ACA, PL, HPF and MEA. Lateral cortical network is comprised of MOp, primary motor area; SSp, primary somatosensory area; VPM, ventral posteromedial nucleus of the thalamus, and ACA.

## Discussion

In this study, we found that both male and female adolescent SR-/- displayed increased social interaction with novel conspecific mice, compared to WT mice. We examined male mice further because of the immediate increase in social exploratory behavior. In male SR-/- mice, there was a rapid decrease in interaction with the familiar mouse in the second trial of the repeat social interaction test and reduced anxiety-related exploratory elevated plus maze paradigm. We show an increase in social novelty-induced cFos network density in the SR-/- compared to WT across the 196 brain regions analyzed in male mice. There was an increase in connectivity in the default-mode network and a decrease in the salience network in the SR-/- mice compared to WT. Overall, our phenotype in adolescence is similar to models of William-Beuren syndrome that display sociability disinhibition and less anxiety (41).

The increased social novelty behavior we observed in the adolescent SR-/- mice is similar to adolescent Grin1 hypomorph mice with near-complete loss of Grin1 expression (42). Adolescent (PND 34–38) Grin1 hypomorph male mice showed a considerable increase in social novelty behavior (42), while adult mouse models of NMDAR hypofunction display social deficits (13, 43, 44). Our previous studies have shown that adult SR-/- mice display NMDAR hypofunction, including reduced NMDAR currents and NMDAR induced postsynaptic potential or current and neurotransmission (15–21). These data suggest that changes in social behavior in SR-/- mice could be through changes in NMDAR activity. However, further investigation is needed to exclude any NMDAR-independent mechanisms during adolescence.

Our results show that SR-/- adolescent mice display a disinhibited exploratory behavior in the EPM paradigm. These effects were not due to an increase in locomotion in the SR-/- male mice. D-serine has been found to play a critical role in regulating glutamatergic neurotransmission, neuronal excitability, and synaptic plasticity in the prefrontal cortex (PFC) via modulating dopamine action at D_1_-type and D_3_-type receptors (21). Modulation of dopamine receptors was able to regulate effects of NMDAR hypofunction on impulsive behaviors (45). Furthermore, juvenile stroke-prone spontaneous hypertensive rats (SHRSP/Ezo) display NMDAR dysfunction and abnormal d-serine metabolism in the mPFC. Administration of D-amino acid oxidase (DAAO, d-serine degrading enzyme) inhibitor rescued attention-deficit/hyperactivity disorder (AD/HD) symptoms (46). NMDAR hypofunction, a hallmark of the SR-/- model, has also been linked to increased exploratory and novelty seeking (47, 48). Lastly, D-serine is important for fear learning (49). In future studies, it would be important to determine if SR-/- mice display risk-taking or impulsive behavior. We used cFos activation correlations between brain areas as a means to predict functional connectivity in the social, salience, lateral cortical, and default mode networks similiar to functional magnetic resonance imaging (fMRI) in humans. We observed increased cFos network density and dysfunctional connectivity (**Table 1**) within human networks and social regions in SR-/- male mice. In multiple networks, there was more cFos coactivation between the anterior cingulate, hippocampal formation regions, and other subcortical regions (**Table 1**). These data are supported by human studies and NMDAR-hypofunction models in mice. Administration of phencyclidine, an NMDAR antagonist, increased activation of frontal-hippocampal regions and the functional connectivity within thalamic networks using EEG and fMRI (50). Resting-state fMRI in adult mice exposed to human recombinant monoclonal NR1 antibody *in utero* showed a reduction in hippocampal functional connectivity (51). In humans, the administration of ketamine (NMDAR antagonist) alters the connectivity of the default mode and salience networks (52) and increases connectivity between thalamic and sensory regions in healthy individuals (53). Patients with schizophrenia who have the disease-associated GRIN2A (NMDAR subunit) variant exhibit increased connectivity in striato-pallido-thalamic regions (54). We observed an increase in connectivity in the default-mode network and a decrease in the salience network in the SR-/- mice compared to WT (**Table 1**). Interestingly, in the SR-/- brains, we observed less cFos correlations in the amygdala (BLA, CEA) and VTA and more in the cortical (ACA), hippocampal, nucleus accumbens areas, which are important for social cognition and reward (**Table 1**). This data suggests that SR deletion may disrupt functional brain networks. However, future studies will be needed to determine if SR deletion alters anatomical connections between areas involved in these networks.

Our study highlights the importance of D-serine availability for proper social behavior and neural connectivity. Future studies exploring the contribution of cell-specific D-serine signaling to NMDAR function, synaptogenesis, excitatory/inhibitory (E/I) balance, and neuronal connectivity will enhance our understanding of the molecular mechanisms of the development of social cognition. More studies are also needed to understand social behavior in SR female adolescent and adult mice.

## Data availability statement

The original contributions presented in the study are included in the article/**Supplementary Material**. Further inquiries can be directed to the corresponding author.

## Ethics statement

The animal studies were approved by McLean Hospital Institutional Animal Care and Use Committee. The studies were conducted in accordance with the local legislation and institutional requirements. Written informed consent was obtained from the owners for the participation of their animals in this study.

## Author contributions

SEB: Data curation, Writing – review & editing, Writing – original draft, Visualization, Methodology, Investigation, Formal analysis, Conceptualization. EN: Writing – review & editing, Visualization, Supervision, Methodology, Formal analysis, Conceptualization. ZW: Writing – review & editing, Writing – original draft, Visualization, Methodology, Formal analysis, Data curation. EE: Writing – review & editing, Visualization, Supervision, Funding acquisition. SB: Writing – review & editing, Supervision, Funding acquisition. DB: Writing – review & editing, Supervision, Funding acquisition, Conceptualization. OF: Visualization, Funding acquisition, Writing – review & editing, Writing – original draft, Supervision, Methodology, Investigation, Formal analysis, Data curation, Conceptualization.

## Supplementary material

Supplementary Figure 1 SR+/- mice display similar social interaction behavior to WT mice. **(A**, **E)** Graphs show the sociability preference indexes. **(B, F)** Graphs show the time in seconds spent interacting with the stimulus mouse and the novel object in the sociability trial. (**C**, **G)**. Graphs show the social novelty preference indexes and the results of one-sample t-tests comparing the observed means to a hypothetical mean. **(D, H)** Graphs show the time in seconds spent interacting with the novel mouse and the familiar mouse in the social novelty trial. Males: N = 7-14; females: N = 5-6. * p < 0.05, ** p < 0.01. One-sample t-test, unpaired t-test and ANOVA.

Supplementary Figure 2 No significant difference in exploratory behavior in adolescent SR+/- mice compared to WT or SR-/-. **(A)** Graph shows the ratio of time spent in the open arms of an elevated plus maze (EPM). **(B)** Graph shows the number of entries to the open arms of the EPM. **(C)** Graph shows the latency in seconds before the first entry to the open arms. **(D)** Graph shows the distance traveled in the maze. N = 10 - 13. * p < 0.05. ANOVA.

Supplementary Figure 3 cFos densities within default-mode, salience, social, lateral cortical network. Graphs show average cFos density in brain areas within the **(A)** Default-mode network **(B)** Salience network **(C)** Social network **(D)** Lateral cortical network. WT: N = 6, SR-/-: N = 5. * p<0.05, q<0.1. Unpaired t-test with Welch correction and FDR correction. Data is presented as Mean ± SEM.
