# Supplementary figures and images for: Serine racemase deletion alters adolescent social behavior and whole-brain cFos activation
Source: Front Psychiatry. 2024 Jun 24;15:1365231. doi: 10.3389/fpsyt.2024.1365231 (PMC11228300; doi:10.3389/fpsyt.2024.1365231)

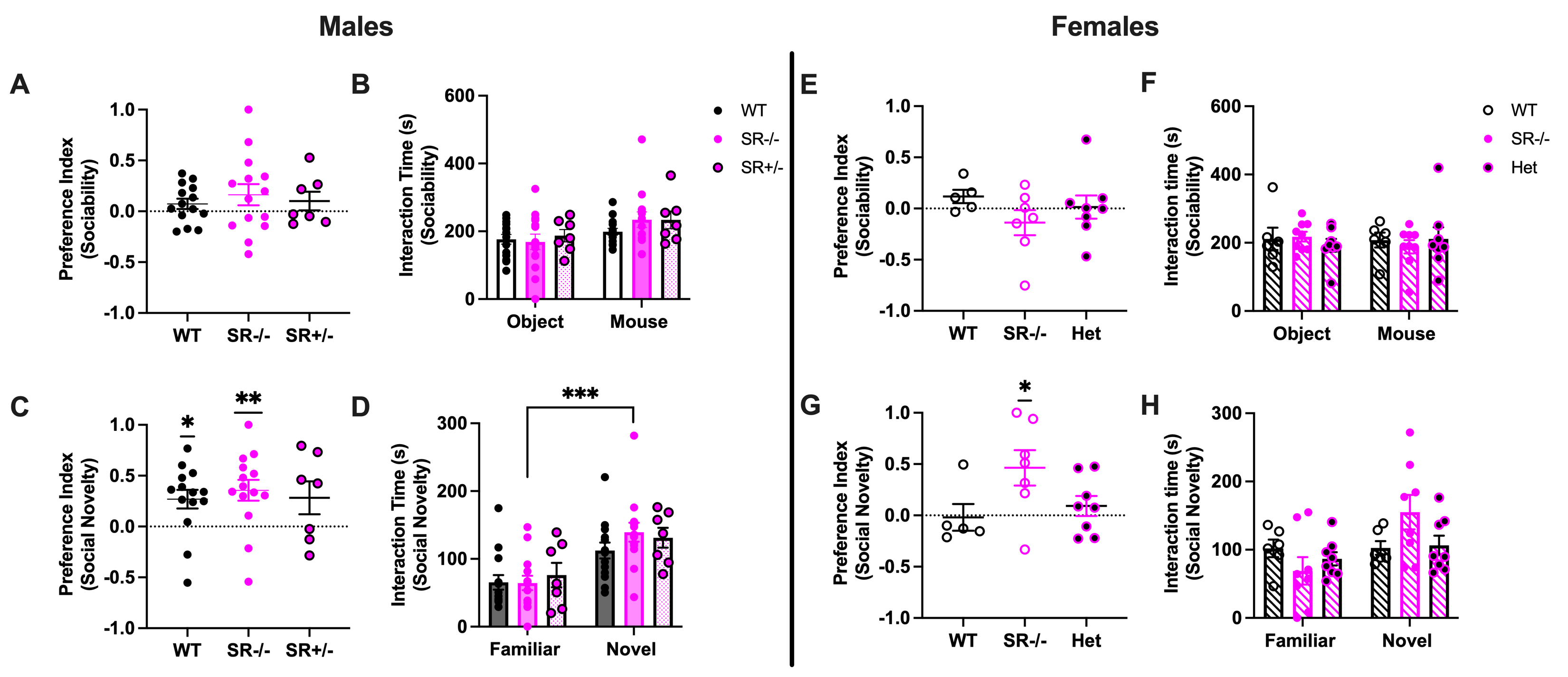

Supplement: Supplementary Figure 1 — SR+/- mice display similar social interaction behavior to WT mice. (A, E) Graphs show the sociability preference indexes. (B, F) Graphs show the time in seconds spent interacting with the stimulus mouse and the novel object in the sociability trial. (C, G). Graphs show the social novelty preference indexes and the results of one-sample t-tests comparing the observed means to a hypothetical mean. (D, H) Graphs show the time in seconds spent interacting with the novel mouse and the familiar mouse in the social novelty trial. Males: N = 7-14; females: N = 5-6. * p < 0.05, ** p < 0.01. One-sample t-test, unpaired t-test and ANOVA. [file Image_1.tiff]

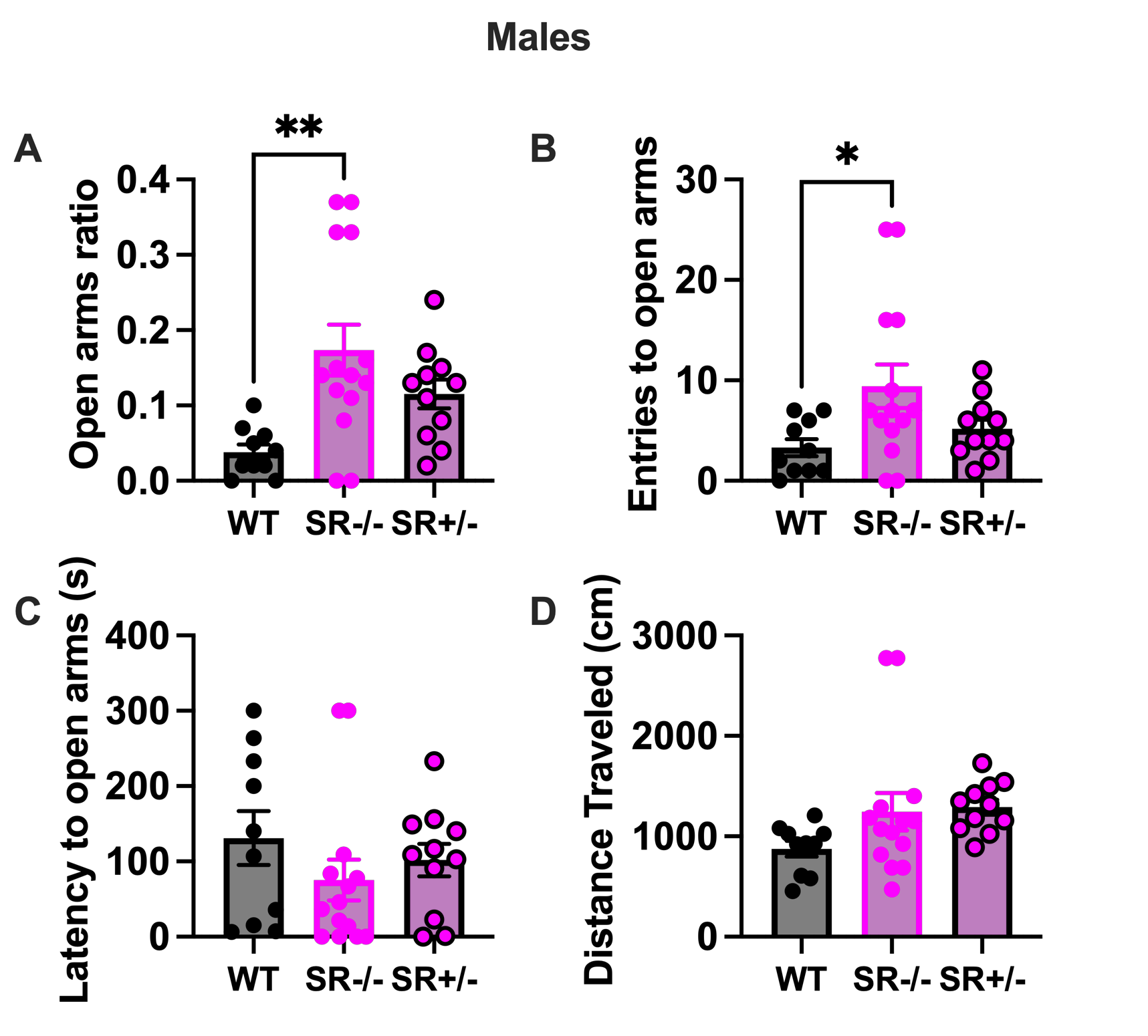

Supplement: Supplementary Figure 2 — No significant difference in exploratory behavior in adolescent SR+/- mice compared to WT or SR-/-. (A) Graph shows the ratio of time spent in the open arms of an elevated plus maze (EPM). (B) Graph shows the number of entries to the open arms of the EPM. (C) Graph shows the latency in seconds before the first entry to the open arms. (D) Graph shows the distance traveled in the maze. N = 10 - 13. * p < 0.05. ANOVA. [file Image_2.tiff]

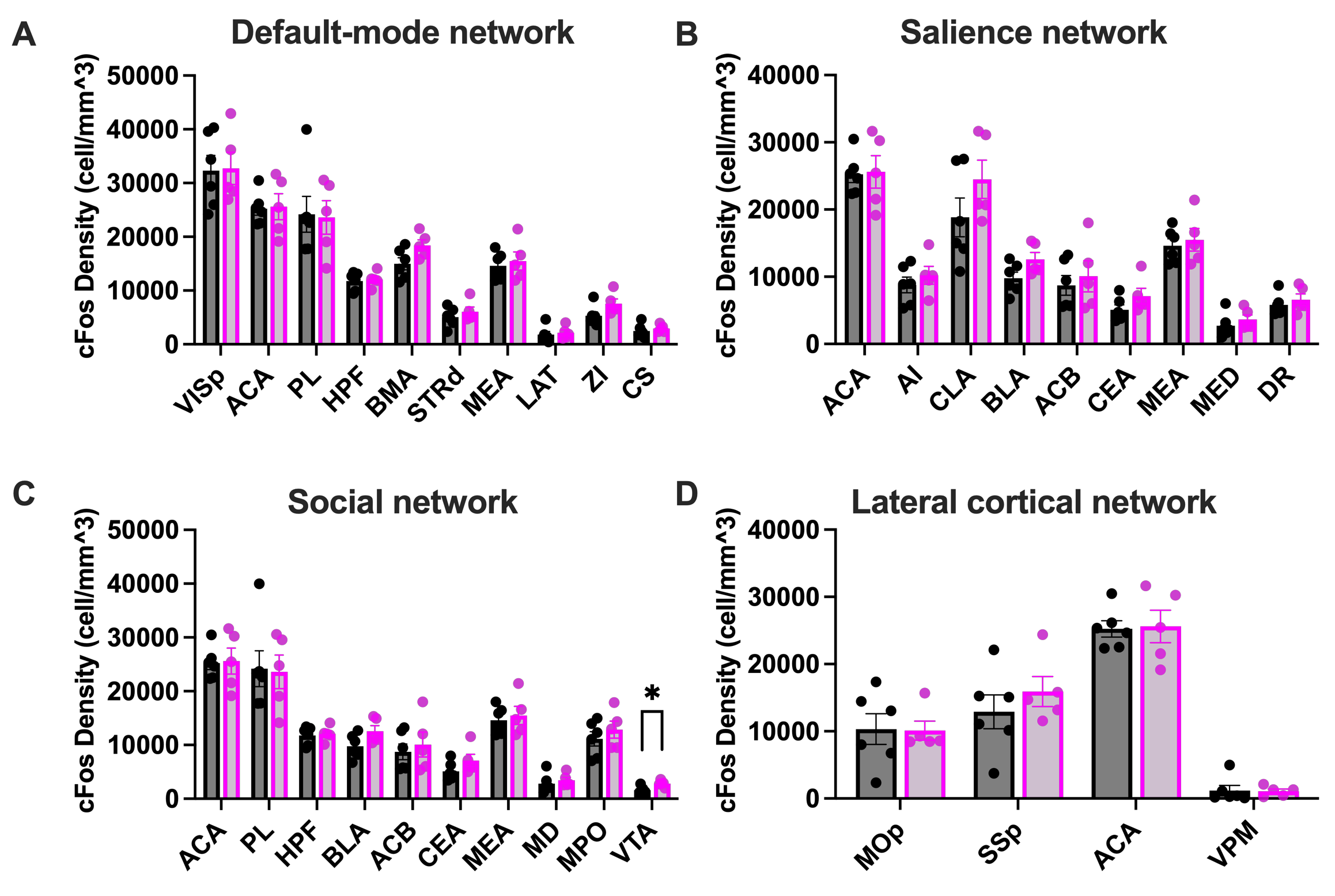

Supplement: Supplementary Figure 3 — cFos densities within default-mode, salience, social, lateral cortical network. Graphs show average cFos density in brain areas within the (A) Default-mode network (B) Salience network (C) Social network (D) Lateral cortical network. WT: N = 6, SR-/-: N = 5. * p<0.05, q<0.1. Unpaired t-test with Welch correction and FDR correction. Data is presented as Mean ± SEM. [file Image_3.tiff]
